# A unit process log reduction database for water reuse practitioners

**DOI:** 10.1016/j.wroa.2024.100226

**Published:** 2024-05-08

**Authors:** Sam Arden, Kyle McGaughy, James Phillips, Linda Hills, Emelyn Chiang, Savana Dumler, Xin ⁽Cissy⁾ Ma, Michael Jahne, Jay Garland

**Affiliations:** a Eastern Research Group, Inc. (ERG), Concord, MA, USA; b United States Environmental Protection Agency, Center for Environmental Solutions and Emergency Response, Cincinnati, Ohio USA

**Keywords:** Non-potable reuse, Log reduction value, Log reduction credit, Human health protection

## Abstract

Pathogen reduction for the purpose of human health protection is a critical function provided by water reuse systems. Pathogen reduction performance potential is dependent on a wide range of design and operational parameters. Poor understanding of pathogen reduction performance has important consequences—under treatment can jeopardize human health, while over treatment can lead to unnecessary costs and environmental impacts. Documented pathogen reduction potential of the unit processes that make up water reuse treatment trains is based on a highly dispersed and unstructured literature, creating an impediment to practitioners looking to design, model or simply better understand these systems. This review presents a database of compiled log reduction values (LRVs) and log reduction credits (LRCs) for unit processes capable of providing some level of pathogen reduction, with a focus on processes suitable for onsite non-potable water reuse systems. Where reported, we have also compiled all relevant design and operational factors associated with the LRVs and LRCs. Overall, we compiled over 1100 individual LRV data entries for 31 unit processes, and LRCs for 8 unit processes. Results show very inconsistent reporting of influencing parameters, representing a limitation to the use of some of the data. As a standalone resource, the database (included as Supplemental Information) provides water reuse practitioners with easy access to LRV and LRC data. The database is also part of a longer-term effort to optimize the balance between human health protection, potential environmental impacts and cost of water reuse treatment trains.

## Highlights

• LRV and LRC data are spread over a wide and variable literature.
• Efficient access to data is a hurdle to its use by water reuse practitioners.
• We present a database of LRVs and LRCs for common water reuse unit processes.
• Results aid in easier data use, performance estimation, and system optimization.
• Better data reporting would improve unit process performance characterization.

## Graphical abstract

**Figure:**
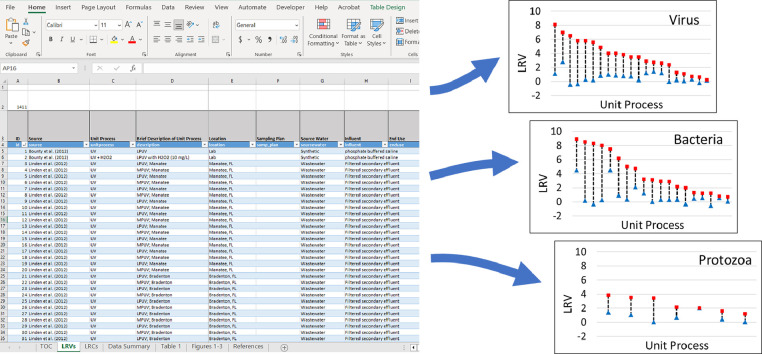

## Public health protection in onsite water reuse

Public health treatment requirements for smaller decentralized or onsite water reuse systems are undergoing a paradigm shift from presumptive to risk-based criteria. Although many existing regulations are still based on end-of-pipe criteria that presume a sufficient level of human health protection (e.g., NSF/ANSI 350, see U.S. EPA, 2021), there is growing consensus that a quantitative risk-based framework is more protective and transparent (NASEM, 2016; NWRI, 2021; Sharvelle et al., 2017; WHO, 2016). Still, there is uncertainty in risk-based treatment targets, as well as our ability to quickly and confidently characterize the performance of treatment processes to meet those targets.

Risk-based treatment targets are expressed as log reduction targets (LRTs), which represent the required cumulative removal of virus, protozoa or bacteria on a log_10_ scale. LRTs are determined by modeling the probability of infection under relevant exposure scenarios using a process called quantitative microbial risk assessment (QMRA) (Jahne et al., 2023; WHO, 2016; Zhiteneva et al., 2020). Many calculated LRTs typically correspond to a 1 in 10,000 risk of infection per person per year (Regli et al., 1991; NWRI, 2021) and, to remain conservative given the variability in pathogen concentrations and exposure pathways, represent the 95th percentile result of the QMRA (Schoen et al., 2017). This means that achieving the LRT for a given pathogen should achieve the benchmark annual infection rate of 1 in 10,000 people 95 % of the time.

Unit processes that make up water reuse treatment trains must provide a cumulative level of pathogen removal, expressed in terms of a log reduction value (LRV), that meets or exceeds the applicable LRT. LRVs are typically demonstrated through extensive (and expensive) validation monitoring (WRF, 2023) or through the use of log reduction credits (LRCs). An LRC is a conservative representation of the LRV for a unit process that is designed and operated within a specific operating envelope, or pre-defined ranges of important design and operational parameters (broadly referred to here as “influencing factors”). The 5th percentile of a specific LRV distribution has become the de facto definition of an LRC. Although less costly to achieve in the short term, using a default LRC approach for system design vs. system-specific validation or challenge testing can potentially result in a system providing greater pathogen removal than it is credited for. Although not a problem from a human health perspective, greater treatment generally comes at the expense of greater inputs of infrastructure, energy, materials, and/or chemicals, which have consequences for system cost and environmental impact (U.S. EPA, 2021b).

From the perspective of water reuse practitioners or researchers, knowing a unit process LRV performance potential—and its influencing factors—of the myriad unit processes that could be used in a water reuse treatment train is critical to the effective design and implementation of these systems. However, the literature on pathogen removal performance is vast and variable, representing a hurdle to those seeking to implement or optimize these systems. Notable reviews exist for single unit processes (e.g., Branch et al., 2021), but a database of multiple unit processes is lacking (Cherchi et al., 2017; Zhiteneva et al., 2020).

To this end, we have compiled LRVs, LRCs and influencing factors for water reuse unit processes into a publicly available Excel database that is included here as Supplemental Information. This database is one part of a longer-term effort to connect human health protection with environmental impacts and costs associated with water reuse treatment trains. However, as a standalone resource, it provides water reuse researchers and design engineers with easy access to LRV and LRC data and their references. The database is not intended to be prescriptive; rather, it is intended as a resource for water reuse practitioners to facilitate easier data access and use. Additionally, the database is not intended to be exhaustive; rather, it includes a review of cornerstone LRC documents and a review of unit process LRV documents that are often cited and broadly relevant to the water reuse community. The remainder of this paper discusses database development, lessons learned, and future research needs.

## Database development

Risk-based treatment guidance, and the data upon which it is based, has evolved somewhat organically through contributions from states, industry working groups, trade publications, and peer-reviewed publications. Rather than perform a traditional literature review based on keyword searches and content screening, we instead compiled LRVs and LRCs from primary sources we, as water reuse practitioners, commonly use or see used within the field (e.g., Collivignarelli et al., 2017; Mosher et al., 2016; Nappier et al., 2018; NASEM, 2016; Pecson et al., 2017; Sharvelle et al., 2017; Soller et al., 2018; Tchobanoglous et al., 2015). Additionally, we used a “snowballing” approach, whereby we used references from our original list of sources to screen for additional data.

Initially, LRV and LRC data were compiled for any unit process that can provide some degree of microbial removal. As the project progressed, our focus narrowed to unit processes likely to be used in onsite non-potable water systems (ONWS), as defined in the National Water Research Institute's (NWRI) On-Site Treatment and Reuse of Nonpotable Water Technical Guidance (NWRI, 2021). These include membrane bioreactors, membrane filters, cartridge filters, and disinfection processes including UV, chlorine, chloramine and ozone. While this narrowing was done to better inform specific research efforts our project team has planned for the near-term, we see this database and its framework being of interest to practitioners outside the ONWS field and have therefore included all data compiled to date, including processes typically included in conventional centralized wastewater treatment facilities.

For all LRCs, dose-response relationships and influencing parameters were compiled. For all LRVs, process characterization parameters were compiled where available. These include process size or capacity metrics, influent water quality, microorganism type and microorganism variability. Although we initially intended for identification of a performance parameter(s) (e.g., membrane pore size for membranes, dosage for disinfection processes) to be a minimum criterion for inclusion in the database, we eventually removed this criterion given the number of studies that did not include these data. For some potential uses of the database, such as determining the optimal dosage of chlorine to achieve an LRT while minimizing cost or environmental impact, the lack of this dosage parameter is a limitation. However, knowing only unit process type, LRV and study citation may still be useful for practitioners interested in seeing the full range of documented LRV performance for a given process and who can perform future investigations into relevant performance parameters. A full list of process characterization parameters can be found in the SI database.

Within the database, efforts were made to qualify LRVs that may have been influent limited with appropriate data flags. For example, in a study of a full-scale plant in Alberta, Qiu et al. (2015) found low removal/inactivation of viruses by chlorination due to the effectiveness of the preceding unit processes and therefore lack of detectable viruses in the chlorination influent (Qiu et al., 2015). The database includes details, when reported, on source water and pathogen concentration to give some context to the removal values presented.

A quality control protocol was followed for all data entry. The database contains the initials of the staff that entered the data, as well as the staff that checked that data entry. In some cases, two sets of initials are input in the quality control columns. Early versions of the database did not include influent and effluent microorganism concentrations and water quality information. After addition of these parameters, an additional round of data entry and review was needed for previously completed entries.

## Database content summary

In total, the database contains LRCs for 8 unit processes across 789 entries and LRVs for 31 unit processes across 1150 entries. Each entry is a specific combination of LRC/LRV and treatment conditions. For LRCs, this means each entry is a unique point within an available dose-response curve, often across variable treatment conditions (e.g., chlorine dose-response across variable temperature and pH). For LRVs, given the variety of experimental methods and reporting approaches, each entry represents either a single sample or a single composite value. LRVs were compiled at the finest resolution available, with composite approaches documented where possible. For the LRV portion of the database, disinfection processes make up the majority of the entries (e.g. 353 entries for UV-based processes, 140 for chlorine disinfection, 64 for ozone) followed by membrane bioreactors (202 entries).

Table 1 provides an overview of the unit processes for which LRCs exist or have been defined, along with their ranges, influencing factors, and comparable LRVs from the literature. A comparison between LRVs and LRCs is intended to illustrate conditions that lead to better or worse LRV performance compared to predefined LRCs. For example, the virus LRC range for MBRs is 1–1.5 log, whereas LRVs as high as 8.7 have been documented. A review of operational or experimental conditions that led to these higher LRVs could help practitioners optimize unit process design and operation.

**Table 1:** Unit processes with log removal credits alongside log removal values and influencing factors.

**Unit Process**	**Influencing Factors**	**LRCs (min-max)**			**LRVs (min-max[n])**		
		**V**	**P**	**B**	**V**	**P**	**B**
UV	turbidity, total suspended solids, UVT, pH, temperature	0.5–6	0.5–6	n.d.	−0.4–5.8 [159]	0.1–3.4 [28]	−0.3–8.3 [166]
UV + H2O2		6–6	6–6	n.d.	0.9–4 [23]	0.7–2.2 [3]	0.4–5 [12]
Membrane bioreactor	Temperature, HRT, SRT, MLSS, membrane fouling and cleaning, membrane ageing and failures	1–1.5	2–2.5	4–4	1.1–8.7 [143]	1.4–6 [13]	3.4–7.5 [46]
Chlorine	turbidity, temperature, pH, disinfection demand	1–4	0–3	1–4	−0.4–6.5 [83]	0.4–0.7 [2]	0.3–8 [55]
Ozone	pH, turbidity, TSS, inorganic ozone demand, dissolved organic content, temperature	1–4	0.25–3	1–4	0.2–5.6 [30]	2–2 [14]	0.3–4 [20]
Microfiltration or Ultrafiltration	transmembrane pressure, temperature, turbidity	n.d.	4–4	n.d.	0.3–5.8 [24]	n.d.	1.5–7.4 [24]
Reverse Osmosis and Nanofiltration	membrane type, permeate flux, cross-flow velocity, recovery, pH, temperature, ionic strength, DOC	2–4	2–4	2–4	2.4–3.9 [33]	n.d.	0–1.9 [28]
Secondary Activated Sludge	SRT	2–2	2–2	n.d.	3.9–3.9 [1]	n.d.	n.d.

*V=virus, *P*=protozoa, *B*=bacteria, n.d.=no data, UV=ultraviolet radiation, H2O2=hydrogen peroxide, UVT=UV transmittance, TSS=total suspended solids, HRT=hydraulic retention time, SRT=solids retention time, MLSS=mixed liquor suspended solids, DOC=dissolved organic carbon (-#) is used to note a negative value. [n] for LRV's denotes number of entries in the database in total for that unit process-pathogen combination.

Compared to LRVs, LRCs come from a small number of sources. The Australian WaterVal program has several validation protocols for determining LRCs for common processes including as chlorination, MBRs, ozone, and reverse osmosis and nanofiltration (WaterSecure, 2017a, 2017b, 2017c, 2017d). The U.S. EPA has guidance documents for UV (US EPA, 2006) and membrane filters (U.S. EPA, 2005), though the guidance for membrane filters only recommends a validation protocol, not actual LRCs. The Water Research Foundation recently released a validation protocol for MBRs (Salveson et al., 2021) that builds on the WaterVal protocol (WaterSecure, 2017b) through incorporation of new data from U.S. MBR studies. Compared to WaterSecure (2017b), the virus LRC from Salveson et al. (2021) decreases from 1.5 to 1 but the protozoa LRC increases from 2.0 to 2.5. Additionally, a number of unit processes have been credited in one-off instances (e.g., Olivieri et al., 2016).

Microorganisms investigated in LRV literature were fairly split between virus and bacteria at 593 and 471 entries, respectively. Protozoa were less common and account for only 86 entries; split evenly between UV, chlorine, and ozone disinfection processes. MS2 bacteriophage and adenoviruses were the most common viruses while *Escherichia coli*, enterococci, and coliforms were the most common bacteria. Protozoa data mostly included *Giardia* and protozoa surrogates; although they are bacteria, we included *Clostridium perfringens* and aerobic spores as protozoa given their potential use as surrogates for protozoan pathogens (Hijnen et al., 2000; WaterSecure, 2017b; WHO, 2017).

**Fig. 1:**
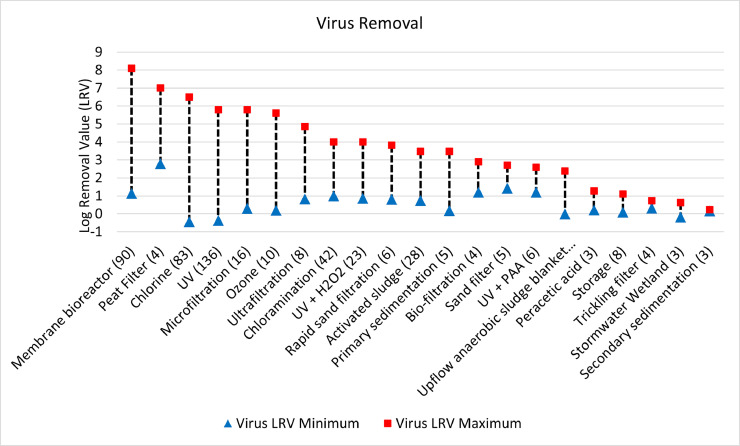
Log removal value (LRV) ranges for virus across all unit process with 3 or more data entries.

**Fig. 2:**
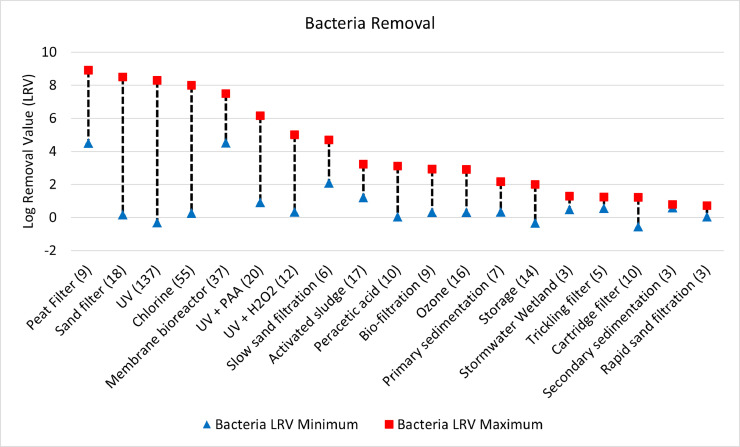
Log removal value (LRV) ranges for protozoa across all unit process with 3 or more data entries.

**Fig. 3:**
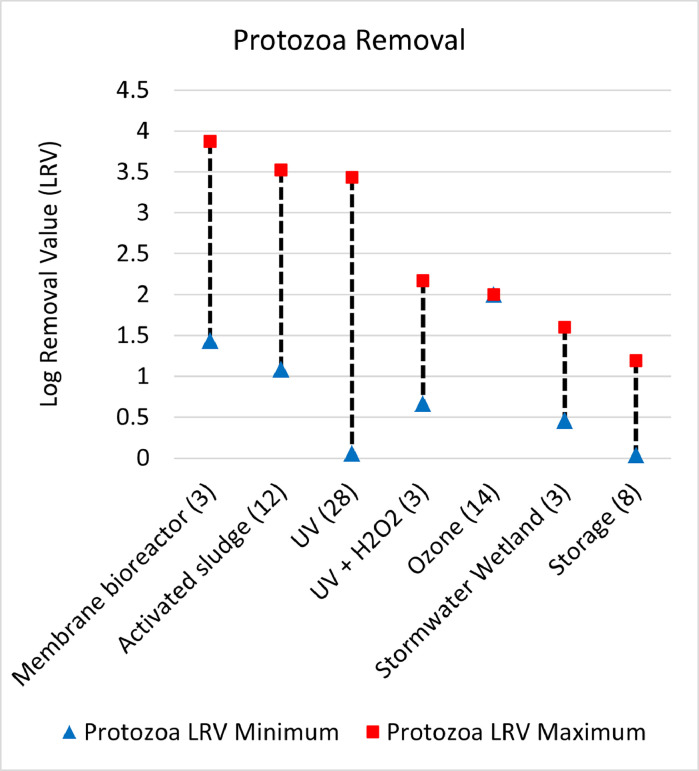
Log removal value (LRV) ranges for bacteria across all unit process with 3 or more data entries.

Fig. 1, Fig. 2, Fig. 3 show the range of LRV entries that were compiled for the majority of unit process (i.e., those with at least 3 entries) currently included in the database. Because this review is not exhaustive and reporting approaches within studies vary, calculating an average value across all entries for each unit process would be inappropriate. Results are provided instead in terms of the minimum and maximum entry for each unit process/pathogen group combination, sorted from highest to lowest maximum entry. The number on each unit process name indicates the number of entries in the database. Several unit processes had results reported where no removal or negative removal was achieved. These results are still useful to the overall database as they give an indication of the conditions (or pathogen type) under which the unit process may be ineffective. These lower bounds were reported in lab, pilot, and full-scale studies and were not just the product of lab scale testing for the lowest point of measurable removal of the target pathogens.

## Discussion and future research recommendations

The Water Research Foundation recently released guidance on developing and implementing study plans to validate LRVs and/or assign LRCs to wastewater treatment systems (WRF, 2023). The guidance includes a recommendation that studies should report performance monitoring parameters and operational factors that affect pathogen removal. While compiling this database, it was observed that much of the existing LRV literature lacks performance characterization data necessary for informing system optimization, system crediting, or for simply improving the mechanistic understanding of process function. Depending on the unit process, these data may include influent water quality data, process design or operational parameters, or microorganism variability. The influencing factors listed in Table 1 provide a starting point for important parameters to consider for each unit process. A more thorough reporting of collected data would help practitioners to better leverage existing data across different studies.

This same finding was echoed in other recently performed review efforts. In a critical review of membrane bioreactor performance, Branch et al. (2021) found “… correlation of MBR operating parameters and LRV was not possible with available literature data, as a full set of operational data was rarely reported.” In a review for investigating the feasibility of defining LRCs for treatment wetlands, Arden et al. (2024) found a similar dearth of operational data reported in that literature.

To define a unit process LRC, researchers must know the distribution of possible LRVs. As LRVs are the difference between influent and effluent concentrations, this means researchers must have knowledge of influent and effluent concentration distributions. While these can easily be calculated by analysis of individual sample data, individual sample data are not always reported. In cases where individual sample data are not reported but mean and standard deviation of the distributions are, guidance is provided in WRF (2023) on how to leverage these statistical parameters directly for estimation of unit process LRCs (i.e., the 5th percentile of the resulting LRV distribution). As can be seen in the corresponding Supplemental Information LRV database, many unit processes without an existing LRC have corresponding LRV studies that do provide influent and effluent concentration mean and standard deviations, which would allow for further exploration of potential LRCs.

Another drawback of the existing LRV literature is its siloed nature. Although human health protection is a fundamental role of water reuse treatment systems, cost and environmental impacts also demand attention if water reuse is to be more widely and sustainably adopted. Specifically, it is possible that the conservative approaches to both setting LRTs and assigning LRCs results in systems with higher than necessary costs and environmental impacts. A simultaneous linking of process design and operation parameters to LRV performance, cost and environmental impact (i.e., through integration with ISO 14,040/14,044 compliant life cycle impact assessment modeling (ISO 2006a, ISO 2006b)) could help identify the potential for system overdesign and advance our knowledge of triple bottom line sustainability. Some work has started exploring the relationship between LRVs and cost (e.g., Linden et al., 2012; Trussell et al., 2015) and even the relationship between all three factors (Arden et al., 2021, 2020; Bhatt et al., 2023), though true optimization work remains lacking.

As this was not an exhaustive review of the literature, additional database development would be a useful contribution to the water reuse community, especially given the rapidly evolving state of the field. While we do not have any immediate plans to continue database development, the database provides a useful framework for others to add to.

## Conclusions

This database is intended to be a tool that water reuse practitioners can use to facilitate future research and ultimately improve the design and operation of ONWS. While not exhaustive, we hope it provides a useful reference to stakeholders in both the practitioner and researcher communities. As always, additional work is needed. This includes more detailed reporting of process design and operational data, measurement of influencing parameters alongside LRV data, and additional research to connect and optimize the linkages between human health protection, environmental impacts and cost to ensure implementation of safe and sustainable water reuse systems.

## Disclaimer

Information has been subjected to U.S. EPA peer and administrative review and has been approved for external publication. Any opinions expressed in this paper are those of the authors and do not necessarily reflect the official positions and policies of the U.S. EPA. Any mention of trade names or commercial products does not constitute endorsement or recommendation for use.

A database reviewing literature with LRV data for various unit processes that can be used for onsite non-potable water systems is included in Excel format.

## CRediT authorship contribution statement

**Sam Arden:** Writing – original draft, Methodology, Investigation, Formal analysis, Data curation, Conceptualization. **Kyle McGaughy:** Writing – review & editing, Methodology, Investigation, Formal analysis, Data curation, Conceptualization. **James Phillips:** Writing – review & editing, Formal analysis, Data curation. **Linda Hills:** Writing – review & editing, Formal analysis, Data curation. **Emelyn Chiang:** Writing – review & editing, Formal analysis, Data curation. **Savana Dumler:** Writing – review & editing, Formal analysis, Data curation. **Xin ⁽Cissy⁾ Ma:** Writing – review & editing, Supervision, Resources, Project administration, Methodology, Funding acquisition, Conceptualization. **Michael Jahne:** Writing – review & editing, Supervision, Resources, Project administration, Methodology, Funding acquisition, Conceptualization. **Jay Garland:** Writing – review & editing, Supervision, Resources, Project administration, Methodology, Funding acquisition, Conceptualization.

## Declaration of competing interest

The authors declare that they have no known competing financial interests or personal relationships that could have appeared to influence the work reported in this paper.

## Data Availability

The database is available as SI and from the EPA ScienceHub at https://doi.org/10.23719/1,529,754 following publication.

## Appendix Supplementary materials

Image, application 1
